# Elbow-sideWINDER (Elbow-side Wearable INDustrial Ergonomic Robot): design, control, and validation of a novel elbow exoskeleton

**DOI:** 10.3389/fnbot.2023.1168213

**Published:** 2023-07-12

**Authors:** Daegeun Park, Christian Di Natali, Matteo Sposito, Darwin G. Caldwell, Jesus Ortiz

**Affiliations:** Advanced Robotics, Istituto Italiano di Tecnologia, Genoa, Italy

**Keywords:** wearable robots, elbow exoskeleton, assistive device, benchmarking and evaluation, electromyography, motion capture

## Abstract

Musculoskeletal Disorders associated with the elbow are one of the most common forms of work-related injuries. Exoskeletons have been proposed as an approach to reduce and ideally eliminate these injuries; however, exoskeletons introduce their own problems, especially discomfort due to joint misalignment. The Elbow-sideWINDER with its associated control strategy is a novel elbow exoskeleton to assist elbow flexion/extension during occupational tasks. This study describes the exoskeleton showing how this can minimize discomfort caused by joint misalignment, maximize assistive performance, and provide increased robustness and reliability in real worksites. The proposed medium-level control strategy can provide effective assistive torque using three control units as follows: an arm kinematics estimator, a load estimator, and a friction compensator. The combined hardware/software system of the Elbow-sideWINDER is tested in load-lifting tasks (2 and 7 *kg*). This experiment focuses on the reduction in the activation level of the biceps brachii and triceps brachii in both arms and the change in the range of motion of the elbow during the task. It is shown that using the Elbow-sideWINDER, the biceps brachii, responsible for the elbow flexion, was significantly less activated (up to 38.8% at 2 *kg* and 25.7% at 7 *kg*, on average for both arms). For the triceps brachii, the muscle activation was reduced by up to 37.0% at 2 *kg* and 35.1% at 7 *kg*, on average for both arms. When wearing the exoskeleton, the range of motion of the elbow was reduced by up to 13.0° during the task, but it was within a safe range and could be compensated for by other joints such as the waist or knees. There are extremely encouraging results that provide good indicators and important clues for future improvement of the Elbow-sideWINDER and its control strategy.

## 1. Introduction

Musculoskeletal disorders (MSDs) are injuries that impact the movement of the body/limb or the musculoskeletal system (i.e., muscles, bones, joints, tendons, ligaments, nerves, discs, and blood vessels), and the most common form of work-related injuries. The elbow joint is involved in many, perhaps even most occupational tasks, and is frequently injured as a result. INAIL (2022) reported that in Italy in 2021 over 11.4% of all work-related MSDs affected the elbow. For elbow-related MSDs, the most commonly reported disorder is elbow tendinopathy (usually called tennis or Golfer's elbow). This disorder is mainly caused by carrying heavy loads and repeated elbow bending, not only for sports players but also for manual workers (Burgess, 1990; Jobe and Ciccotti, 1994; Verhaar, 1994). In addition, Rineer and Ruch (2009) mentioned that tendon rupture is also a critical disorder. Although tendon rupture is less frequent than elbow tendinopathy, it results in a more significant disability and loss of function. The main cause of the tendon rupture is a sudden, eccentric contraction of the biceps, frequently happening when holding or lifting loads during occupational tasks. To prevent or reduce these elbow disorders, there are several effective strategies, such as distribution of loads or impacts on the elbow, mechanical stabilization of the elbow joint, and restriction of elbow movement within the range of motion (ROM).

To further enhance safety and reduce risk, several devices have been developed, with some having been commercialized. These range from elastic elbow braces to exoskeletons (Gull et al., 2020). The elastic elbow brace is especially widely used to stabilize the elbow and reduce stress (Hattori et al., 2017). However, due to its simple structure, the brace cannot play a role in reducing muscle fatigue or preventing over-extension. To move beyond this, many researchers have developed exoskeletons to actively assist the elbow, but there are significant challenges in moving to employ these in industrial applications. In industrial tasks, there is a growing use of passive shoulder exoskeletons that generate assistive forces as reported by Voilqué et al. (2019). Although shoulders are anatomically complex joints, passive shoulder exoskeletons need to consider only simple joint kinematics between the torso and the shoulder, and it is relatively easy to estimate the assistive torque. For elbows, however, the required assistive torque is influenced by two joints: the shoulder and the elbow. For example, when the worker is holding a load in their hand with an elbow flexion of 90°, the elbow torque needed to support the load against gravity is different with a shoulder flexion of 0 and 90°. Thus, to generate the assistive torque using a passive mechanism, any elbow exoskeleton must have a much more complex mechanism that considers the simultaneous movements of the shoulder and the elbow. Due to this complexity, active elbow exoskeletons that do not suffer from this joint–joint coupling are often preferred. Most of these prototypes employ simple mechanical structures with actuators, not only distributing external loads into the structure but also providing the assistive force according to various arm movements.

Although active elbow exoskeletons can consequently be less complex than passive devices, there are still significant design challenges that reduce the usability of the exoskeleton in real worksites. The challenge is to address the complexity of the human skeleton. Human joints commonly consist of a combination of complex skeletal structures, and the elbow joint is a complex gliding hinge joint composed of three bones as follows: the humerus, radius, and ulnar. This complexity makes it difficult to align the elbow with a single revolute joint, i.e., the structure that forms the most common mechanical joint in robot designs. Any misalignment between the elbow and the assistive device can cause not only discomfort but also damage to the joint or skin. Many researchers have proposed various design approaches to compensate for joint misalignment. One emerging design approach is the Soft Exosuit (Xiloyannis et al., 2022). The Soft Exosuit consists of a fabric-based elbow brace and flexible force transmission. Owing to the flexibility of the structure, the Soft Exosuit can easily adapt to any complex human joints while having a compact structure. Several Soft Exosuits for the elbow pull the forearm toward the upper arm to generate the assistive torque using pneumatic actuators (Kobayashi and Hiramatsu, 2004; Abe et al., 2019) or tendon-driven mechanisms (Koo et al., 2014; Steven et al., 2018; Xiloyannis et al., 2019). Although these Soft Exosuits are compact and light, because of the lack of rigidity, it is difficult for them to compensate for the force that compresses the user's joint when pulling the actuators or tendons toward the joint. This compression results in a limit in the applicable assistive force and usability. On the other hand, some other Soft Exosuits assist the elbow without the compression issue. Thalman et al. (2018) propose a balloon-like soft cylindrical actuator that pushes the forearm away from the upper arm to flex/extend the elbow. With this pushing approach, there is little risk that the elbow joint is inappropriately compressed by the exoskeleton. However, the comparatively large size of the actuator and the possibility of compressing the skin and twisting the joint due to the flexible structure must be solved for safety and efficiency.

A second design approach that aligns the joints is to use a traditional mechanical structure that fully constrains the degrees of freedom (DoFs) of the elbow joint (Cempini et al., 2013; Schorsch et al., 2022). This approach has the advantage of protecting the elbow from undesired forces and efficiently transmitting the assistive force. However, due to the weight and size of the mechanism, there is a limitation in using the exoskeleton in real worksites, especially in a narrow or confined space. In this study, to improve usability by balancing joint constraint and flexibility, we have developed a self-alignment mechanism that decouples the elbow rotation from its translation. Stienen et al. (2009) also considered the self-alignment mechanism for the elbow joint based on joint decoupling. In their mechanism, the rotation of the linkage that attaches to the forearm is operated by the actuator, while the translation of the linkage is freely allowed in a passive manner. When the rotation of the linkage assists the elbow flexion/extension, the free translation ensures that the actuation joint is passively aligned with the elbow joint. By selectively constraining the DoF of the elbow, the exoskeleton with the decoupling mechanism can be more compact and lighter than the fully constrained one.

**Figure 1:**
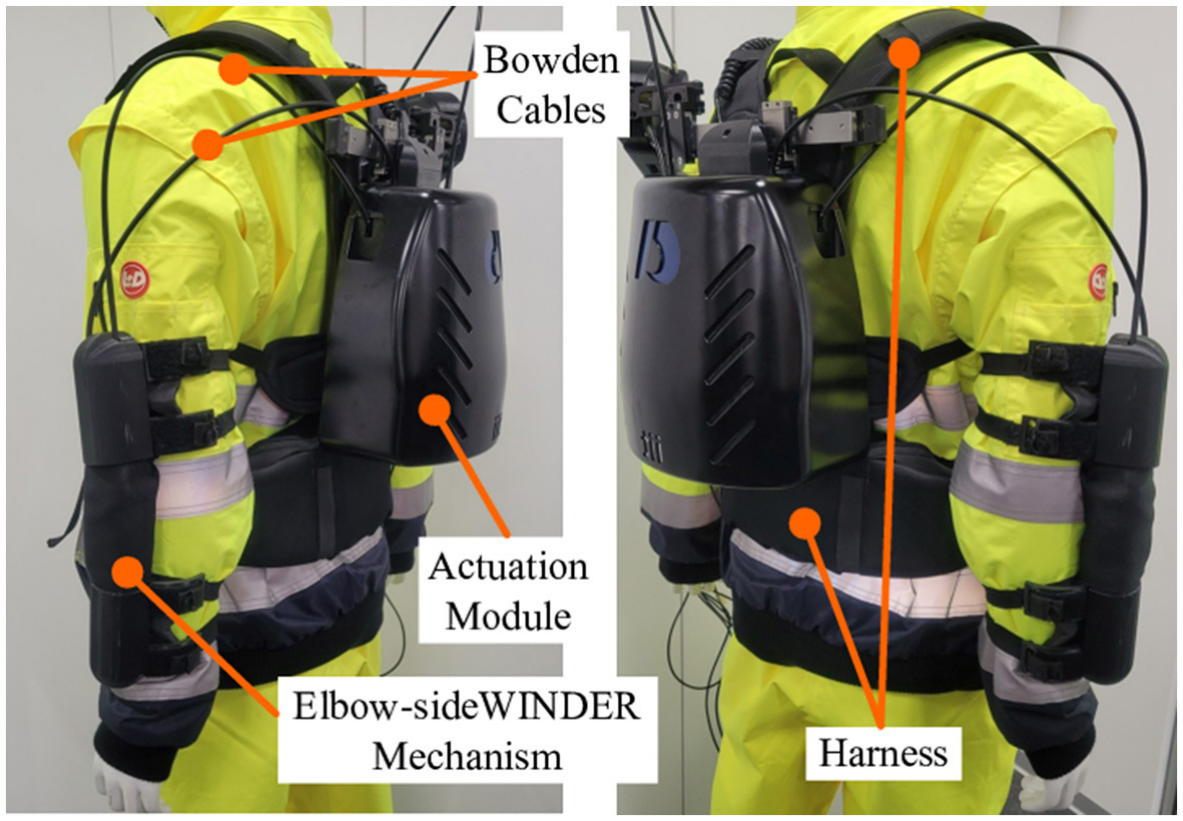
Elbow-sideWINDER: elbow exoskeleton consists of Joint Alignment Mechanism, Actuation Module, Bowden Cables, and Harness.

In this study, we propose the Elbow-sideWINDER (Figure 1) that builds on the decoupling mechanism presented (Stienen et al., 2009) and its associated control strategy. The control strategy has been designed based on our previous study (Park et al., 2022a) that controls the shoulder exoskeleton, the Shoulder-sideWINDER (Park et al., 2022b). The cutting-edge control strategies, usually developed for rehabilitation (Proietti et al., 2016; Qian et al., 2022), require a fully constrained joint mechanism and a number of sensors and actuators that should be equal to or exceed the number of joints. However, due to several critical factors such as the high cost, weight, and complexity of the system, the control strategies for such devices are of limited value in real industrial applications. To design a control strategy that meets industry requirements, we categorize controllers into three levels. The first level is a high-level controller (HLC) that classifies a task based on the user's intent and sets the parameters based on the user's physical characteristics. For example, when a user holds and moves a drill, the HLC detects and classifies whether the user intends to lower the drill, push the drill toward a wall, or perform some other actions. Based on the intended task and user's characteristics, the second-level controller, the medium-level controller (MLC), calculates the required torque corresponding to the dynamics of the arm. The dynamics of the arm are measured by physical sensors, such as an IMU while performing the task. The third stage is a low-level controller (LLC) that controls the actuator to generate the target force/torque calculated by the MLC. In this study, our control strategy focuses on the MLC that effectively generates the assistive force according to dynamic arm movements and changes in the external loads. This is achieved through the use of minimal sensors and low computational burden. To satisfy this consideration, the MLC uses a single sensory system based on Myo Armband [MYO, a commercial surface electromyography (sEMG) with eight sEMG sensors and one 3-axis accelerometer, Thalmic Labs, United States]. The 3-axis accelerometer integrated within the MYO is used in an arm kinematics estimator that determines the assistive torque according to the dynamic movements of the arm. The sEMG sensors in the MYO are used in a load estimator that estimates the external load to calculate the assistive torque.

In addition to presenting the combined hardware/software system of the Elbow-sideWINDER, this study assesses the capabilities of the system in industrial applications. The target task assessed is specified as a real logistic scenario of lifting and lowering a box using the left and right arms simultaneously. The effectiveness of assistance is assessed using the activation levels of two muscles, the biceps brachii caput brevis and the triceps brachii caput lateral head, while lifting, holding, and lowering a load. The kinematic change due to the exoskeleton is also assessed using the change in the ROM of the elbow measured by a motion capture system.

This study, comprehensively, presents the design of the Elbow-sideWINDER in Section 2.1, and its control strategy and experimental protocol are presented in Section 2.2. The validation results are presented in Section 3. Finally, this study is discussed and concluded in Section 4.

## 2. Materials and methods

### 2.1. Elbow-sideWINDER: a novel elbow exoskeleton for industrial applications

The Elbow-sideWINDER has been designed to assist with elbow flexion/extension in industrial applications. This exoskeleton consists of two novel mechanisms to improve usability in real worksites. One is a joint alignment mechanism to compensate for the joint misalignment between the user's anatomical joints and the exoskeleton, and the other is an actuation mechanism to reduce the size and inertia around the upper arm.

#### 2.1.1. Joint alignment mechanism

**Figure 2:**
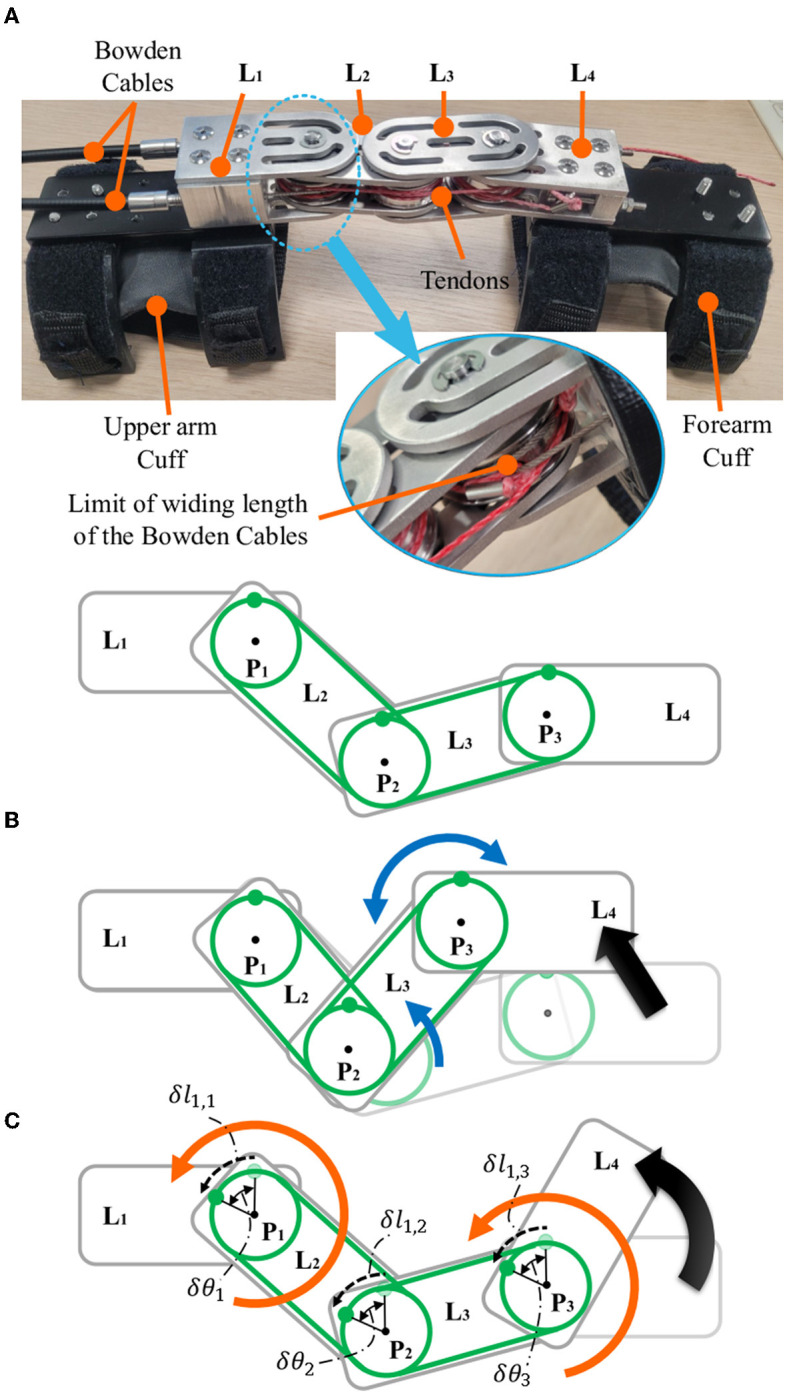
Elbow-sideWINDER prototype **(A)** and schematics of the translation **(B)** and the rotation **(C)** of *L*_4_. The green lines indicate the tendons, *T*_1_ and *T*_2_. The blue and orange arrows indicate the movement of linkages and pulleys, respectively.

Anatomically, the elbow joint is a glide-hinge joint, so the center of rotation (CoR) of the elbow changes in a passive manner according to the flexion angle. To align any exoskeleton's joint with the user's movable joint, there should be a joint alignment mechanism that focuses on decoupling the rotational movement of the exoskeleton from the translational movement. The rotational movement actuated by the actuation module applies the assistive torque in the direction of the elbow flexion/extension, while the unconstrained translation aligns the CoR of the exoskeleton with that of the elbow in a passive manner. To decouple these movements, the Elbow-sideWINDER has a joint alignment mechanism (Figure 2A) that consists of three pulleys (*P*_1,2,3_) with the same radius (*r*), four linkages (*L*_1,2,3,4_), and two tendons (*T*_1,2_). The linkages are connected in series, and each pulley is placed at each linkage connection joint. *L*_1_ and *L*_4_ are attached to the upper arm and forearm, respectively. *P*_1_ is actuated by Bowden cables from the actuation module and can freely rotate with respect to the linkages. *P*_2_ is not rigidly connected to the tendons and any linkages, so it can move freely regardless of the movements of the tendons, other pulleys, and linkages. *P*_3_ is rigidly fixed at *L*_4_. Two tendons wrap *P*_1_-*P*_3_ in opposite directions, such as agonists and antagonists. Each end of the tendons is rigidly anchored to *P*_1_ and *P*_3_. The winding length of *T*_*i*_ at *P*_1_ is equal to that at *P*_3_. This is given as follows:


(1) $$\begin{array}{l} \delta l_{i,1}-\delta l_{i,3}=r\delta \theta_{1}-r\delta \theta_{3}=0{\;}_{\left(i=1,2\right)} \end{array}$$

(2) $$\begin{array}{l} \delta \theta_{1}=\delta \theta_{3} \end{array}$$

where *δl*_*i,j*_ is the change in the winding length of *T*_*i*_ at *P*_*j*_, and *δ*θ_*j*_ is the rotational angle of *P*_*j*_. Because *P*_3_ and *L*_4_ are rigidly connected, the orientation of *L*_4_ is determined by the rotation of *P*_3_.

Based on this model, the rotation of *L*_4_ along with the forearm is only driven by the actuation module and decoupled from the translation of *L*_4_. For example, when *L*_4_ translates with respect to *L*_1_, *L*_2_, and *L*_3_ rotate independently regardless of the rotation of *P*_1_. It compensates for the change in the winding length of *T*_1_ and *T*_2_, as shown in Figure 2B. When *P*_1_ rotates counter-clockwise by winding *T*_1_, *T*_1_ pulls *P*_3_, and hence *P*_3_ and *L*_4_ rotate counter-clockwise together (Figure 2C). Consequently, the actuator rotates the pulley *P*_1_, *P*_1_ winds the tendon *T*_1_/*T*_2_, and *T*_1_/*T*_2_ rotates the pulley *P*_3_, and hence the orientation of *L*_4_, which is rigidly connected to P3 and the forearm, changes. This is all achieved regardless of the translation of the forearm.

For safety, there is an important mechanical constraint on the rotation of *L*_4_. Because the rotation of *L*_4_ is controlled by *P*_1_, the winding length of the Bowden cable that actuates *P*_1_ is limited, as shown in Figure 2. When the connecting point of the Bowden cable reaches the minimum distance between *P*_1_ and the sheath of the Bowden cable, the rotation of *P*_1_ is blocked. Finally, the ROM of *P*_1_ is limited to that of the elbow.

#### 2.1.2. Actuation module

**Figure 3:**
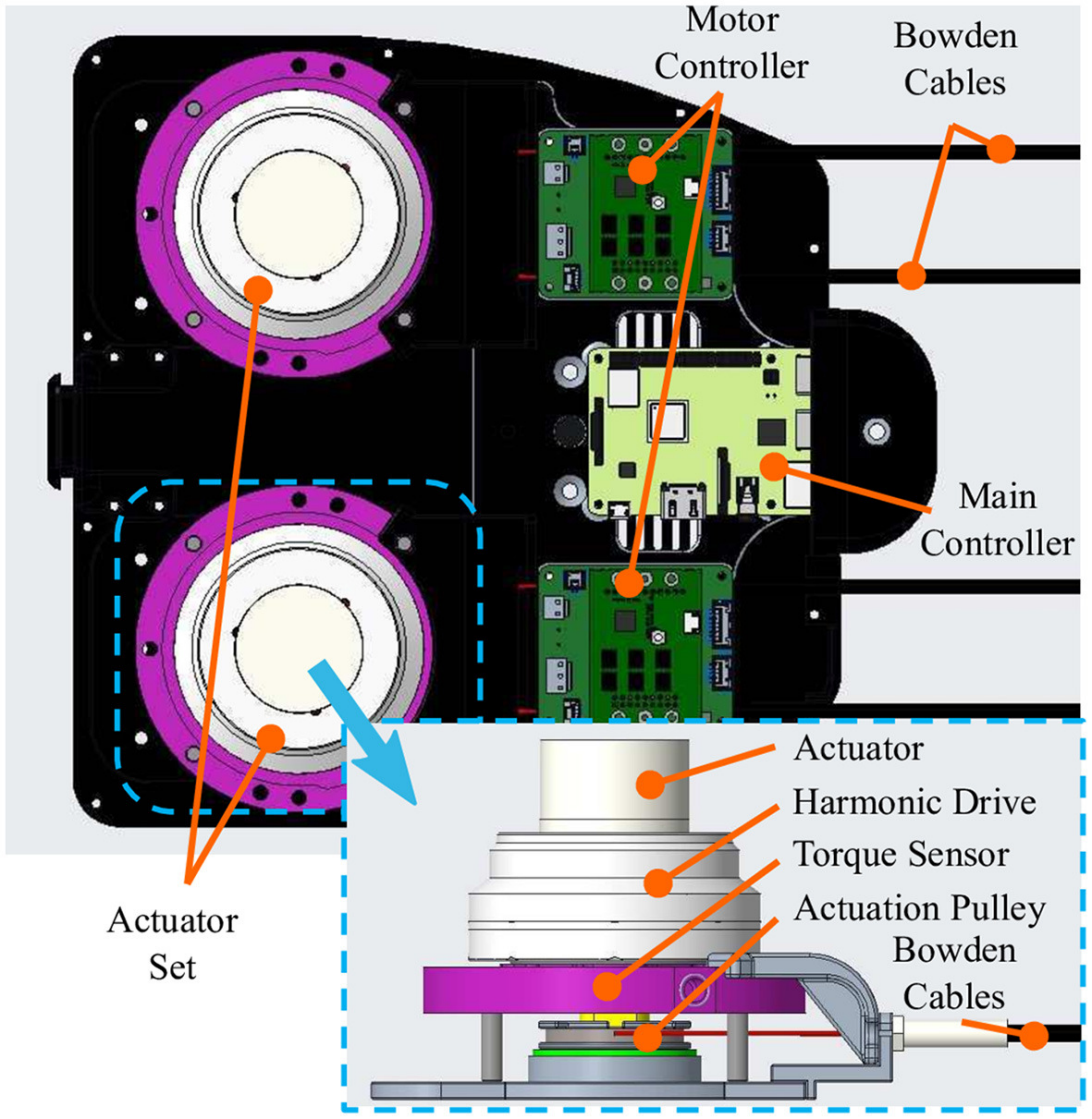
Actuation module with an actuator, a harmonic drive, an actuation pulley, a torque sensor, a motor controller, and two Bowden cables.

In many occupational tasks, the arm is frequently required to dynamically move within a narrow or confined physical space. Thus, designing a compact unit that minimizes the size and inertia is essential in increasing usability in real worksites. The Elbow-sideWINDER employs a tendon-driven mechanism to transmit the assistive force to the elbow, creating minimal size and inertia on and around the arm. As shown in Figure 3, the actuation module for each arm consists of an actuator (EC45, Maxon, Switzerland), a harmonic drive (CSD-17-050, Harmonic Drive, United States), an actuation pulley, a torque sensor (TS110a, ME-Meßsysteme, Germany), a motor controller (Escon 50/5, Maxon), and two Bowden cables. The two Bowden cables work as agonists/antagonists, connect the actuation pulley to *P*_1_ of the Elbow-sideWINDER mechanism, and hence transmit the assistive torque to the elbow. The torque sensor with a 20 *Nm* capacity is used to regulate the LLC that controls the actuation torque with a simple P controller. The main controller (Raspberry Pi 3B+, Raspberry Pi Foundation, UK) runs both the LLC and MLC, recording data from the actuators, sensors, and controllers at a frequency of 100 *Hz*.

### 2.2. System validation

#### 2.2.1. Control strategy

**Figure 4:**
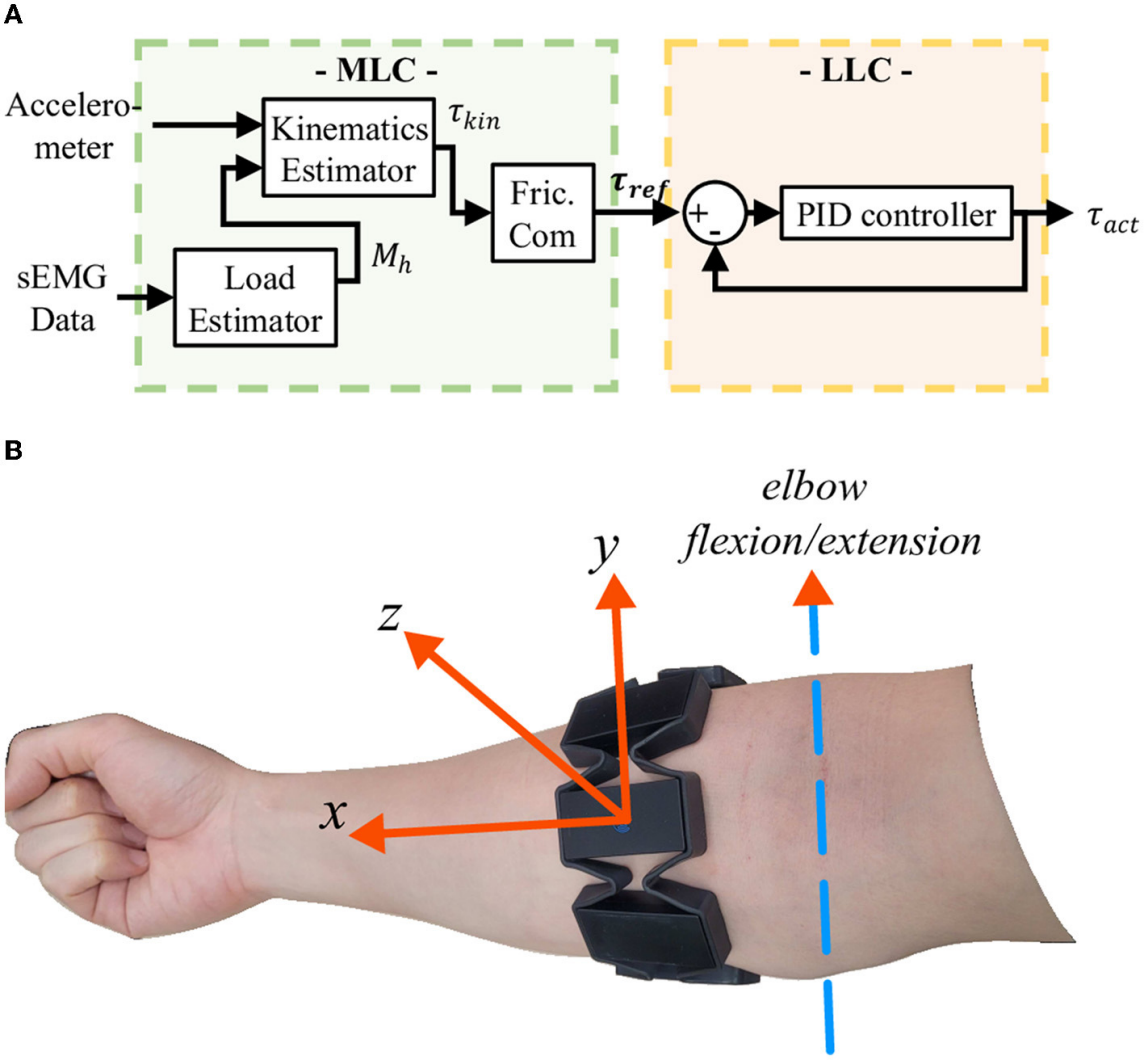
**(A)** Controller block diagram. The MLC consists of three control units as follows: arm kinematics estimator, load estimator, and friction compensator (Fric.Com). The two estimators estimate the required assistive torque based on arm kinematics and an external load being carried in the hand. The friction compensator amplifies the kinematic torque τ_*kin*_ to compensate for the friction on the Bowden Cable. **(B)** Alignment of the accelerometer integrated within the MYO.

To assist with elbow flexion, the Elbow-sideWINDER generates an assistive torque using the MLC, as shown in Figure 4A. The MLC consists of three control units as follows: the arm kinematics estimator, the load estimator, and the friction compensator, using only one sensory system, the MYO. This minimizes the complexity, size, computational burden, and cost of the entire system.

The arm kinematics estimator estimates forearm dynamics using data from the three-axis accelerometer integrated within the MYO. The accelerometer ($\vec{A_{MYO}}$) is mounted so that the x-axis is aligned from the elbow to the wrist, the y-axis is parallel to the rotational axis of the elbow flexion/extension, and the z-axis is perpendicular to the x- and y-axes (Figure 4B). The z-axis value is maximized when the forearm (x-axis) and the rotational axis of the elbow flexion/extension (y-axis) are perpendicular to gravity. Since the target movement for the assistance is elbow flexion/extension, the acceleration at the z-axis represents the action that the exoskeleton assists. Thus, the assistive torque (τ_*assist*_) is equivalent but in the opposite direction to the sum of the torques generated by the forearm mass (τ_*f*_) and an external load (τ_*h*_) being carried in the hand. Each torque is given as follows:


(3) $$\begin{array}{l} \tau_{f}=A_{MYO,z}\cdot M_{f}d_{f} \end{array}$$

(4) $$\begin{array}{l} \tau_{h}=A_{MYO,z}\cdot M_{h}d_{h} \end{array}$$

(5) $$\begin{array}{l} A_{MYO,z}=\vec{A_{MYO}}\cdot {\left[0\;0\;1\right]}^{T} \end{array}$$

(6) $$\begin{array}{l} \tau_{kin}=\tau_{f}+\tau_{h} \end{array}$$

where *A*_*MYO,z*_ is the acceleration at the z-axis, *d*_*f*_/*d*_*h*_ is the center of mass of the forearm or hand, *M*_*f*_ is the mass of the forearm, and *M*_*h*_ is any external load.

The load estimator using the eight sEMG sensors in the MYO estimates *M*_*h*_ which is derived as the average of the normalized muscle activation levels. This is given as follows:


(7) $$\begin{array}{l} M_{h}=K_{myo}\cdot \frac{myoEMG_{avg}}{myoEMG_{max}} \end{array}$$

where *myoEMG*_*avg*_ and *myoEMG*_*max*_ are the average muscle activation level of the eight sEMG sensors and their maximum voluntary contraction (MVC) values, respectively. The MVC values are measured when the user clenches their fist as hard as possible. The control gain, *K*_*myo*_, is set to control the level of assistance and customize the torque according to the external load based on the sEMG characteristics. These characteristics are different for each user. The estimated external load is used to calculate the desired torque. Consequently, the assistive torque (τ_*kin*_) is derived with the gain, *K*_*total*_, that controls the final level of assistance, given as follows:


(8) $$\begin{array}{l} \begin{array}{c} \tau_{kin}=K_{total}\times \left(\vec{A_{MYO}}\cdot {\left[0\;0\;1\right]}^{T}\right)\times \;\left(M_{f}d_{f}+K_{myo}\times \frac{myoEMG_{avg}}{myoEMG_{max}}d_{h}\right). \end{array} \end{array}$$

The friction compensator amplifies the assistive torque to compensate for the torque loss caused by the friction on the Bowden cable. The actuator always pulls the Bowden cable to rotate the forearm, which always generates a positive normal force between the cable and the sheath. Thus, the simplified Capstan friction model is suitable for the friction compensator. The torque transmitted to the elbow (τ_*out*_) is determined by the input torque generated by the actuator (τ_*in*_), the friction coefficient (μ), and the bending angle of the Bowden cable (θ_*b*_), given as follows


(9) $$\begin{array}{l} \tau_{in}=\tau_{out}e^{\mu \theta_{b}}. \end{array}$$

The friction coefficient (μ) is set to 0.126, which was characterized experimentally in the previous study (Park et al., 2022a) because the same Bowden cable is used. The required output torque (τ_*out*_) is the assistive torque (τ_*kin*_), so, finally, the reference actuation torque (τ_*ref*_) is as follows:


(10) $$\begin{array}{l} \tau_{ref}=\tau_{kin}e^{0.126\theta_{b}}. \end{array}$$

The reference torque is controlled by the LLC using a simple P controller with a P gain of 15. Since the friction on the Bowden cable acts as an energy dissipator like a D gain, the D gain is set to 0. The I gain is set to zero because an I gain can diverge the assistive torque when the force transmission is delayed due to tendon elongation. For safety, the maximum assistive torque is limited to 15 *Nm* which is less than the torque sensor capacity.

#### 2.2.2. Experimental setup

**Figure 5:**
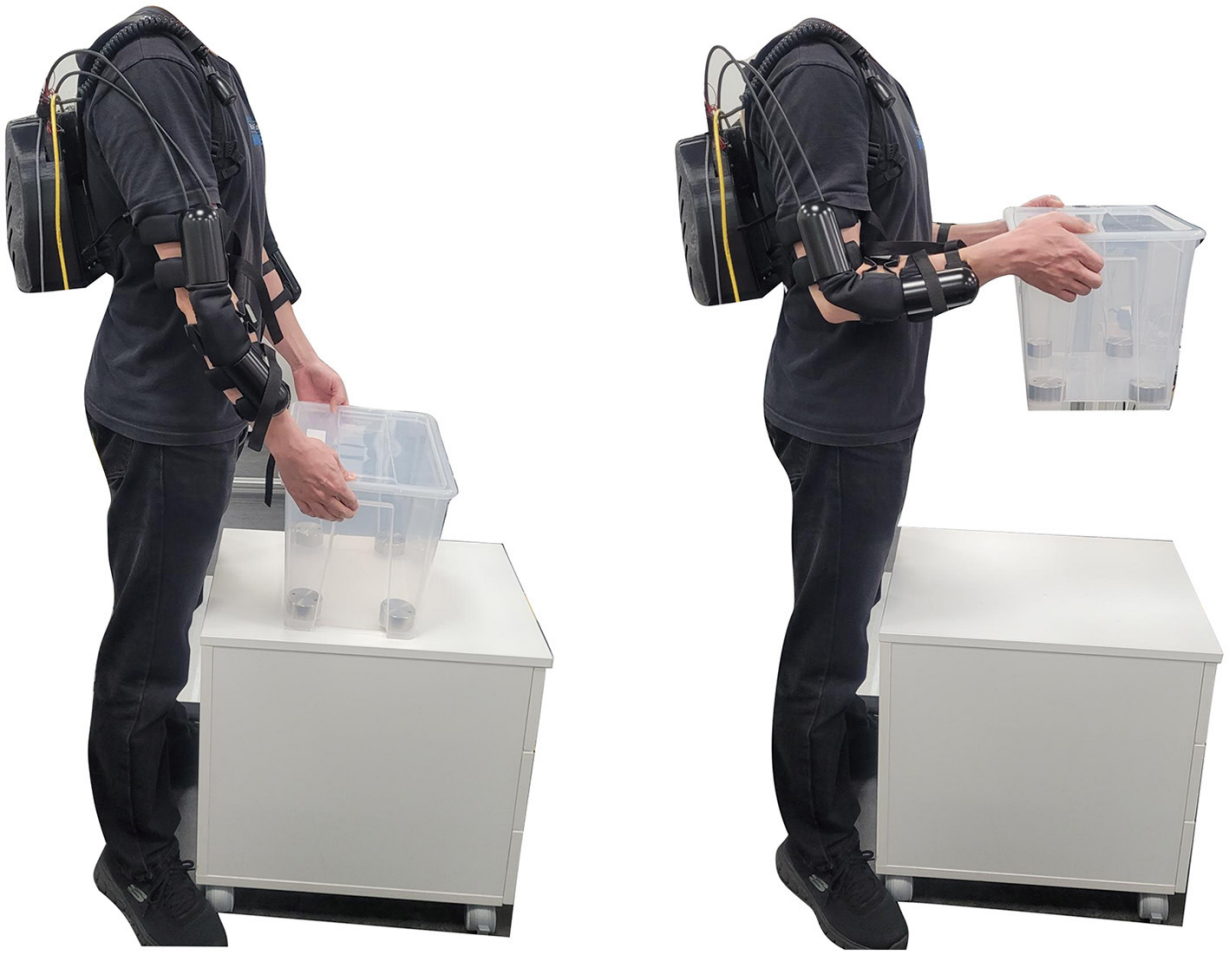
Elbow-sideWINDER experimental protocol. A target load, 2 or 7 *kg*, is contained in a regular box [size: 0.39(*L*) × 0.28(*W*) × 0.28(*H*) *m*^3^] placed on a table at a height of 0.57 *m* that participants could reach without bending their torso. The participants were using a side grasp to lift the box.

The Elbow-sideWINDER mechanism and its control strategy have been validated in a load-lifting task that is commonly performed in logistics. In the experimental protocol, the task is designed to have a worker use both arms simultaneously to lift a box, as shown in Figure 5. There were two target loads (2 and 7 *kg*), and the load was contained in a regular box [size: 0.39(*L*) × 0.28(*W*) × 0.28(*H*) *m*^3^]. The regular box was at a height of 0.57 *m* that participants could reach without bending their torsos. The participants were using a side grasp to lift the box.

To evaluate the effectiveness of the assistance, participants experimented with two device states, wearing and not wearing the exoskeleton. According to the protocol, assistive torques were applied to both arms simultaneously when wearing the exoskeleton. In the MLC, the bending angle (θ_*b*_) of the friction compensator was set to π, since the upper arm was barely elevated and the angles of the Bowden cables were also fixed during the experiment. The control gains, *K*_*total*_ and *K*_*myo*_, were set to 1.

Two measurements are used to validate the assistive performance of the exoskeleton. The first is the activation level of the two target muscles, biceps brachii caput brevis (*Bi*) and triceps brachii caput lateralis (*Tr*), which are responsible for elbow flexion and extension, respectively. The muscle activation level was measured by a commercial sEMG system (FREEEMG, BTS Bioengineering, Italy), with a frequency of 1 *kHz*. The second measurement is the upper arm movement, measured by an IMU-based motion capture system (Xsens MVN, Xsens Technologies B.V., the Netherlands), with a frequency of 60 *Hz*. The motion capture system was worn only on the upper body including the head, the arms, the torso, and the waist.

#### 2.2.3. Experimental protocol

Five healthy persons (gender: two females, three males, age: 28.8 ± 1.8, height: 175.0 ± 5.6 *cm*, weight: 65.4 ± 8.0 *kg*) participated in the validation trials. Before starting the experiment, each participant attached four sEMG electrodes to the target muscles of both arms and wore the motion capture system on the electrodes. The motion capture system was calibrated according to the manufacturer's instructions. To normalize the sEMG signal, the MVC of each muscle was measured in two postures. Each posture involves the participant either pushing their hands downward onto the table or pushing up under the table with an elbow flexion angle of 90°. The participant activated the muscles for 3 *s* and rested for 3 *s*. This was repeated three times in each direction.

After setting up the equipment, the participant stood in a fixed position in front of the box, as shown in Figure 5. Following verbal instructions, the participant lifted and held the box for 5 *s* at an elbow flexion angle of 90°, and then lowered the box and rested for 5 *s*. The participant conducted five sets of lifting-lowering motions per session. This was repeated twice, each in four experimental conditions combining two target loads and two device states. All participants performed the sessions when not wearing the exoskeleton first and then repeated them after wearing the exoskeleton. There was a 5−*min* break between sessions to avoid muscle fatigue. Thus, a total of eight sessions (40 motions) were performed per participant.

#### 2.2.4. Data analysis

**Figure 6:**
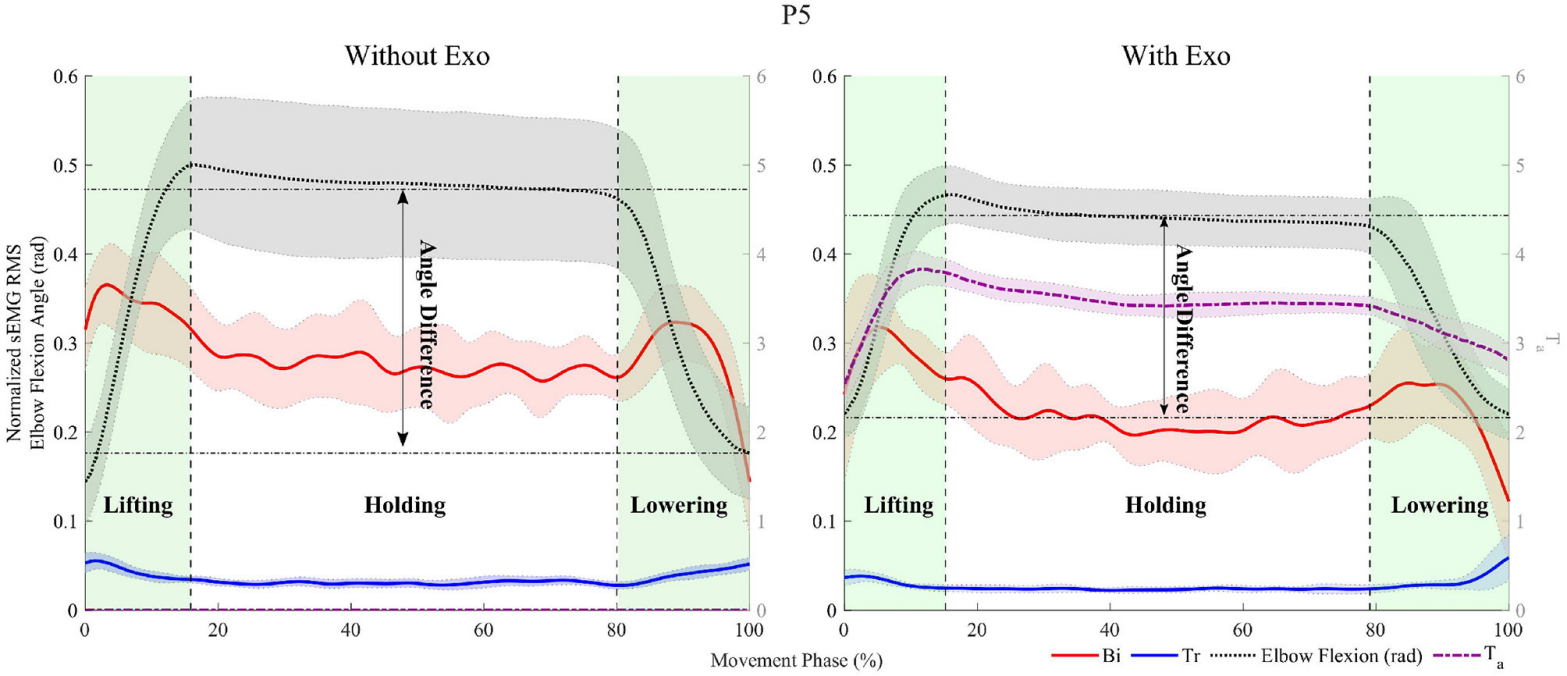
Representative data for participant, *P*5, with a 7 *kg* load. The experimental data are divided into three areas corresponding to the movements, lifting, holding, and lowering. The red, blue, dashed black, and dashed-dot purple lines indicate the moving *RMS* of the normalized sEMG signal of the Biceps Brachii caput brevis (*Bi*) and Tricep Brachii caput lateralis (*Tr*) with 100 samples, the elbow flexion angle (*rad*), and the assistive torque (*Nm*), respectively. The angle difference indicates the difference in the elbow flexion angle between the average angle during holding and the angle immediately after placing the box on the table.

To remove noise, sEMG signals were filtered offline with a fourth-order Butterworth filter (cutoff frequency of 15 and 450 *Hz*) and a notch filter (cutoff frequency of 50 Hz). The muscle activation level was analyzed in three arm motions, lifting, holding, and lowering, referring to the study by Missiroli et al. (2022). The arm motions were determined by the elbow flexion angle measured by the motion capture system, as shown in Figure 6. The root mean square (RMS) of the normalized sEMG signal was calculated for each motion interval for each session and used as an indicator of the effectiveness of the assistance. If the RMS value is greater when wearing the exoskeleton than when not wearing it, this indicates that the exoskeleton reduced the muscle effort during the target motion.

The difference in the elbow ROM from the holding to lowering motions is used as the indicator to evaluate the change in the elbow kinematics when wearing the exoskeleton. The elbow ROM during the task was calculated by subtracting the angle immediately after placing the box on the table from the average angle while holding it. The difference in the elbow ROM was calculated by subtracting the elbow ROM when not wearing the exoskeleton from that when wearing it. If the difference in the elbow ROM is negative, this indicates that the elbow was less extended when wearing the exoskeleton while lowering the box.

All data were statistically analyzed using a *t*-test. The statistical analysis tested the null hypothesis that the value of each indicator when wearing the exoskeleton was not significantly different from when not wearing the exoskeleton. If a *p*-value of the *t*-test between two device states is >0.05, it indicates that there was no significant difference, and the null hypothesis is accepted.

## 3. Results

### 3.1. Muscle activation level

**Figure 7:**
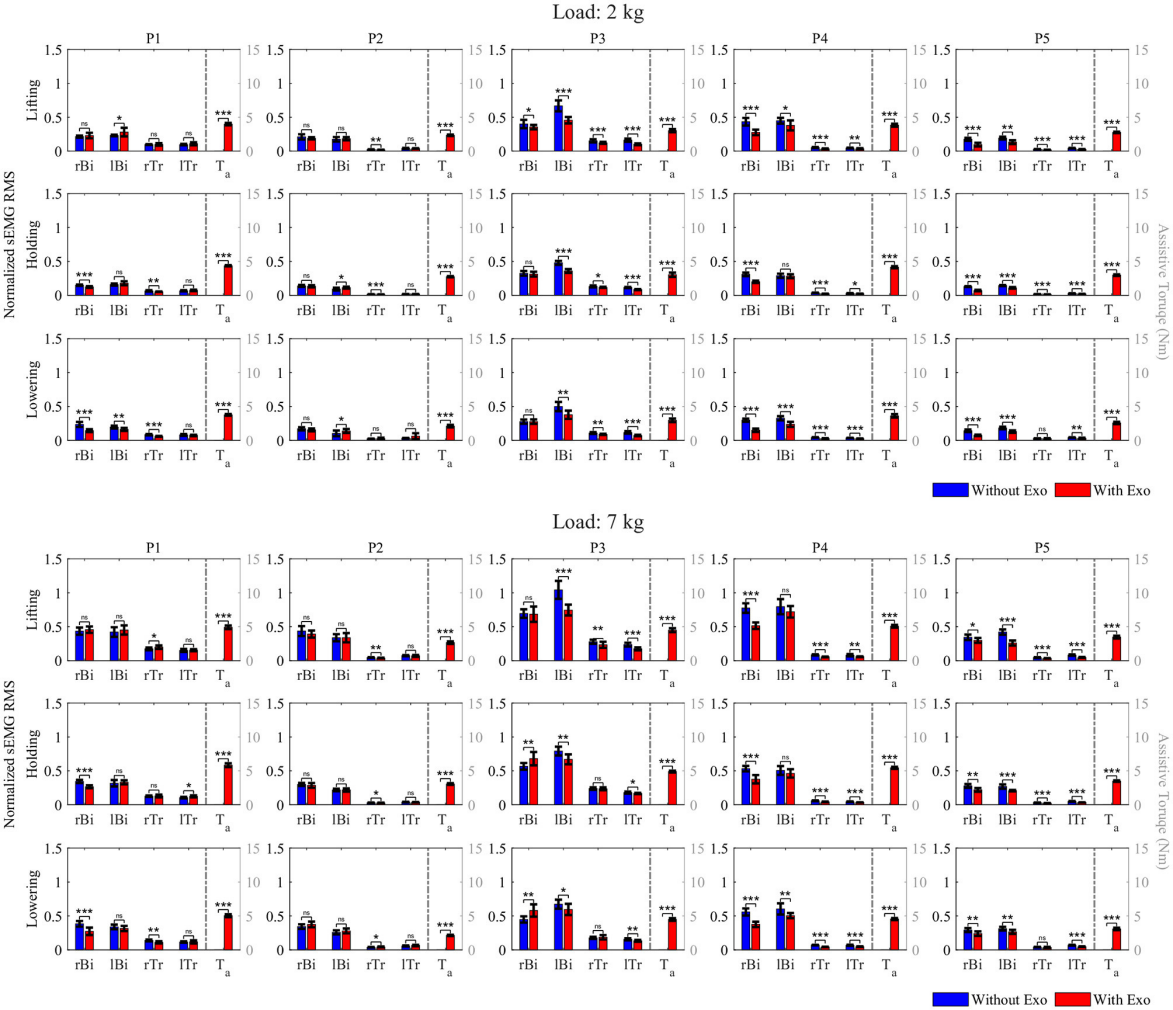
RMS of the normalized activation level of *Bi* and *Tr* in the right (*rBi*, *rTr*) and left (*lBi*, *lTr*) arms during lifting, holding, and lowering, and average assistive torque (*T*_*a*_) while holding a 2 or 7 *kg* load. (^*ns*^*p* > 0.05, ^*^p < 0.05, ^**^p < 0.01, and ^***^p < 0.001).

The assistive performance of the Elbow-sideWINDER was evaluated through a comparison between the RMS values of the normalized activation level of *Bi* and *Tr* in both arms when wearing and not wearing the exoskeleton. The results are shown in Figure 7 and presented in Table 1. Additionally, Table 2 shows the average assistive torque in the three motions.

**Table 1:** Percentage of the reduction in RMS of the normalized activation level of *Br* and *Tr* in both arms.

**Unit (%)**		**2 kg**						**7 kg**					
		**Biceps Brachii (Bi)**			**Triceps Brachii (Tr)**			**Biceps Brachii (Bi)**			**Triceps Brachii (Tr)**		
		**Right**	**Left**	**Avg**.	**Right**	**Left**	**Avg**.	**Right**	**Left**	**Avg**.	**Right**	**Left**	**Avg**.
P1	Lifting	0.0^ns^	−22.1^*^	−11.1	0.0^ns^	0.0^ns^	0.0	0.0^ns^	0.0^ns^	0.0	−14.0^*^	0.0	−7.0
	Holding	17.8^***^	0.0^ns^	8.9	18.4^**^	0.0^ns^	9.2	22.6^***^	0.0^ns^	11.3	0.0^ns^	−17.4^*^	−8.7
	Lowering	39.0^***^	17.1^**^	28.1	29.5^***^	0.0^ns^	14.7	28.8^***^	0.0^ns^	14.4	20.3^**^	0.0^ns^	10.1
P2	Lifting	0.0^ns^	0.0^ns^	0.0	21.5^**^	0.0^ns^	10.8	0.0^ns^	0.0^ns^	0.0	16.4^**^	0.0^ns^	8.2
	Holding	0.0^ns^	−23.1^*^	−11.5	20.7^***^	0.0^ns^	10.4	0.0^ns^	0.0^ns^	0.0	6.7^*^	0.0^ns^	3.4
	Lowering	0.0^ns^	−35.0^*^	−17.5	0.0^ns^	0.0^ns^	0.0	0.0^ns^	0.0^ns^	0.0	−14.1^*^	0.0^ns^	−7.0
P3	Lifting	11.3^*^	31.7^***^	21.5	19.3^***^	37.4^***^	28.4	0.0^ns^	28.6^***^	14.3	16.4^**^	26.2^***^	21.3
	Holding	0.0^ns^	25.2^***^	12.6	11.3^*^	26.5^***^	18.9	−20.1^**^	15.4^**^	−2.3	0.0^ns^	7.0^*^	3.5
	Lowering	0.0^ns^	24.3^**^	12.2	18.1^**^	38.5^***^	28.3	−29.5^**^	11.4^*^	−9.1	0.0^ns^	13.9^**^	7.0
P4	Lifting	36.3^***^	15.3^*^	25.8	36.2^***^	22.3^**^	29.2	33.6^***^	0.0^ns^	16.8	34.9^***^	28.5^**^	31.7
	Holding	35.5^***^	0.0^ns^	17.7	36.5^***^	18.3^*^	27.4	29.2^***^	0.0^ns^	14.6	26.6^***^	22.2^***^	24.4
	Lowering	49.9^***^	27.7^***^	38.8	35.4^***^	28.5^***^	32.0	32.8^***^	16.4^**^	24.6	38.9^***^	31.4^***^	35.1
P5	Lifting	45.1^***^	30.9^**^	38.0	35.5^***^	38.4^***^	37.0	13.0^*^	38.4^***^	25.7	26.2^***^	42.3^***^	34.2
	Holding	45.2^***^	23.6^***^	34.4	29.7^***^	30.6^***^	30.2	20.7^**^	22.5^***^	21.6	23.3^***^	29.3^***^	26.3
	Lowering	47.5^***^	28.8^***^	38.2	0.0^ns^	25.4^**^	12.7	18.3^**^	15.2^**^	16.8	0.0^ns^	32.3^***^	16.1
							**Effect of elbow-sideWINDER:**				**Positive**		**Negative**

The value, *Avg*., is the average for both arms. A positive value (blue cell) indicates reduced muscle activation and a negative value (red cell) indicates increased muscle activation. The zero value indicates that there is no significant difference between wearing and not wearing the exoskeleton (^*ns*^*p* > 0.05,

* *p* < 0.05,

** *p* < 0.01,

*** *p* < 0.001).

**Table 2:** Average assistive torque in the three motions.

**Unit (Nm)**		**2 kg**	**7 kg**
P1	Lifting	4.0	4.9
	Holding	4.3	5.8
	Lowering	3.8	5.0
P2	Lifting	2.3	2.7
	Holding	2.7	3.1
	Lowering	2.1	2.1
P3	Lifting	3.0	4.5
	Holding	3.0	4.9
	Lowering	3.0	4.5
P4	Lifting	3.8	5.1
	Holding	4.1	5.5
	Lowering	3.6	4.5
P5	Lifting	2.8	3.5
	Holding	3.0	3.5
	Lowering	2.6	3.1

For the 2 *kg* load condition, the RMS values of *Bi* activation are always significantly smaller (or zero change) when wearing the exoskeleton for three of the five participants, *P*3–*P*5. For example, for participant *P*5, when wearing the exoskeleton, the activation level of *Bi* in both arms decreased by 45.1% (right) and 30.9% (left) during lifting, 45.2% (right) and 23.6% (left) during holding, and 47.5% (right) and 28.8% (left) during lowering, respectively. For participant *P*1, the results generally showed a reduction (or zero change) in *Bi* activation under all conditions apart from left arm lifting, where there was a 22.1% increase in activation. Exceptionally, for participant *P*2, *Bi* activation showed non-significant change on the right arm for all three sequences and an increase of 23.1 and 35.0% for left arm holding and lowering, respectively.

In the case of *Tr*, the overall data indicate that this muscle never experienced an increased activation in any of the participants while wearing the exoskeleton. Participants *P*3–*P*5 each had a significant reduction in activation of both the left and right arms in all instances except *P*5 right arm lowering (0.0%). For participants *P*1 and *P*2, the right arm activation in most instances was reduced (never increased); however, for both participants, there were a majority of zero change results (0.0%) that indicate no significant difference between the two device states.

For the 7 *kg* load condition, *Bi* was activated significantly less (or zero change) when wearing the exoskeleton under all conditions except for *P*3 right arm holding and lowering. For *P*2, there are no significant differences between the two device states in both arms in all instances. For *P*3, the right *Bi* activation showed significant increases of 20.1 and 29.5% when wearing the exoskeleton in right arm holding and lowering, respectively. However, the increase in muscle activation level in the right arm is similar to the decrease in the left arm, less than ~9.1%.

The RMS values of *Tr* are also always significantly smaller (or zero change) when wearing the exoskeleton for four participants, *P*2–*P*5, apart from *P*2 right arm lowering. For *P*1, when wearing the exoskeleton, *Tr* activation showed increases of 14.0 and 17.3% for right arm lifting and left arm holding, respectively. For *P*2, only the right *Tr* was significantly activated 14.1% more when wearing the exoskeleton during lowering, and there were a majority of zero change results (0.0%) in the left arm in all instances.

### 3.2. Elbow flexion angle

**Figure 8:**
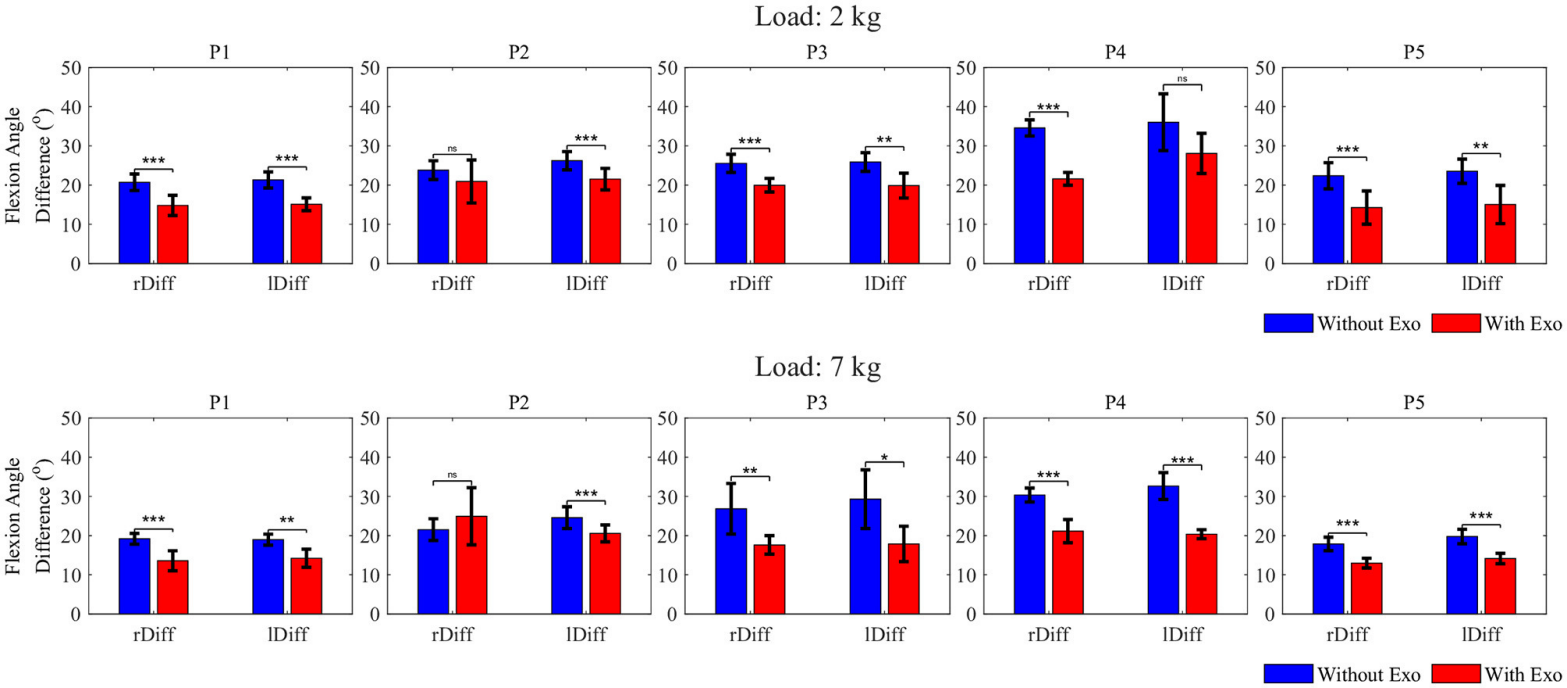
Elbow ROM between the average elbow angle during holding and the angle immediately after placing the box on the table, with a 2 or 7 *kg* load (^*ns*^*p* > 0.05, ^*^p < 0.05, ^**^p < 0.01, and ^***^p < 0.001).

The difference in the elbow ROM is also important in determining the effect of the Elbow-sideWINDER in arm kinematics during occupational tasks. Figure 8 shows the elbow ROM between the average angle during holding and the angle immediately after placing the box on the table when wearing and not wearing the exoskeleton. Table 3 presents the difference in the elbow ROM between both device states.

**Table 3:** Difference in the elbow ROM between the average elbow angle during holding and the angle immediately after placing the box on the table.

**Unit (^o^)**	**2 kg**			**7 kg**		
	**Right**	**Left**	**Avg**.	**Right**	**Left**	**Avg**.
P1	−5.9^***^	−6.2^***^	−6.1	−5.6^***^	−4.7^**^	−5.2
P2	0.0^ns^	−4.7^***^	−2.4	0.0^ns^	−4.0^***^	−2.0
P3	−5.6^***^	−6.0^**^	−5.8	−9.2^**^	−11.4^*^	−10.3
P4	−13.0^***^	0.0^ns^	−6.5	−9.2^***^	−12.3^***^	−10.8
P5	−8.1^***^	−8.5^**^	−8.3	−4.9^***^	−5.6^***^	−5.3

The value, *Avg*., indicates the average for both arms. The negative value indicates that the elbow was less extended when wearing the exoskeleton. The zero value indicates that there is no significant difference between wearing and not wearing the exoskeleton (^*ns*^*p* > 0.05,

* *p* < 0.05,

** *p* < 0.01, and

*** *p* < 0.001).

For both load conditions, when lowering the load, the result shows that at least one elbow was significantly less extended when wearing the exoskeleton than when not wearing the exoskeleton. The maximum reductions in the elbow ROM were ~−8.3° for *P*5 and −10.8° for *P*4, on average, for both arms with loads 2 and 7 *kg*, respectively.

## 4. Discussion

Overall, for both loads, *Bi* responsible for the elbow flexion was significantly less activated in most instances for four (*P*1, *P*3, *P*4, and *P*5) of the participants when wearing the exoskeleton for the three target motions (up to 38.8% with a 2 *kg* load and up to 25.7% with a 7 *kg*, on average for both arms). This result shows that the Elbow-sideWINDER assists the elbow flexion during lifting, reduces muscle effort on *Bi* during holding, and stabilizes the elbow joint during lowering. In addition, the activation level of *Tr* responsible for the elbow extension significantly decreased in most instances for three participants, *P*3, *P*4, and *P*5 when wearing the exoskeleton (up to 37.0% with a 2 *kg* load and up to 35.1% with a 7 *kg*, on average for both arms). This indicates that the exoskeleton is also helpful in the stabilization of the elbow joint performed by *Tr* in all the motions. Although the overall results show that the Elbow-sideWINDER is already very effective in reducing muscle effort with regard to elbow flexion/extension, several considerations will help to further enhance the performance and usability.

First, there is optimizing the control gain in the load estimator (*K*_*myo*_). The result for *P*2 shows increases or no significant difference in *Bi* activation between the two device states in all instances. *Bi* activation also showed increases for *P*1 left arm lifting. These results indicate that the exoskeleton had no effect on or even hindered *Bi*. This negative or non-significant effect may have resulted from insufficient assistive torque due to a non-optimized gain. In fact, for *P*2, the assistive torque was the smallest among all participants, and the effect of the exoskeleton was also the lowest. In (8), the assistive torque corresponding to the external load is smaller for a user who has stronger forearm muscles with a higher MVC value. It means that the stronger the participant's muscles, the less assistive force the MLC generates. Moreover, if the assistive torque generated is less than the mechanical resistance of the exoskeleton, a user with stronger muscles encounters resistance rather than assistance. As a result, less assistive torque in participants with stronger muscles could lead to greater or non-significantly different muscle activation. In addition, when comparing the results between 2 and 7 *kg* loads, the issue of insufficient torque generation occurred even when the external load was changed. The results show that the reduction in muscle activation level was greater for the 2 *kg* than the 7 *kg*. This difference occurred because, in the latter instance, the assistive torque increased insufficiently to counteract the increase in the target load. Table 2 presents that the load increased by a factor of 3.5, while the assistive torque only increased by a factor of 1.6. Due to this insufficient increase in the assistive torque, the participants exerted relatively higher muscle effort for the 7 *kg*. To generate sufficient assistive force, it is necessary to optimize *K*_*myo*_ in real-time, by considering the individual user's muscular characteristics including the MVC of the forearm muscle.

There are, however, various challenges to mapping muscle activation levels with the required force, such as unconscious over-contraction or muscle fatigue. For example, when a worker lifts a load of unknown weight, to control dynamic movements of the elbow, an agonist muscle (e.g., Biceps Brachii) can be often excessively activated and then an antagonist muscle (e.g., Triceps Brachii) can also be more activated than in the normal situation. Muscle activation also increases as muscles become fatigued as reported by Hug et al. (2003), so the mapping between muscle activation level and the assistive torque should change in real time. To take these situations into account, it is necessary to change the gain of the load estimator in real-time, by analyzing not only the muscle condition but also the intended task. In future studies, we are planning a variety of case studies with different gains and loads, to establish a high-level strategy to optimize the gain in the load estimator (*K*_*myo*_). Additionally, the correlation between the reduction in muscle activation level and assistive torque will be identified in these future case studies. In this study, there is no significant correlation between the amount of assistive force and the reduction in muscle activation level. For example, for the 2 *kg* load condition during holding, the maximum assistive torque was 4.3 *Nm* for *P*1, but the maximum reduction in *Bi* occurred in the result for *P*5. Through the case studies with various amounts of torque, a correlation model between the reduction in muscle activation level and the assistive torque will be identified. Finally, the model will be used to determine an optimal assistive torque according to a target task.

The second consideration is balancing assistance between the right and left arms. Comparing the results of the right arm and the left arm, it is frequently observed that *Bi* was less activated in only one arm when wearing the exoskeleton. For example, the result for *P*3 with a 7 *kg* load shows that, when wearing the exoskeleton, the right *Bi* was more activated and the left *Bi* was less activated during holding and lowering. This indicates that the activation of the right and left *Bi*s was not balanced, affecting the performance of the exoskeleton. One reason for the unbalanced muscle activation is the uneven weight distribution of the box. The box was bigger than the size of the loads used, so the weight could move and not be centered on the box during the task. If users feel that the weight of the box is unevenly distributed when lifting, they can contract one arm more than the other to keep the balance. In a future study, one more sensory system such as MYO will be integrated, and the controller will control each arm independently to identify the effect of the balance issue on both hands during cooperation tasks.

Third, the attachment of the exoskeleton to the arm properly is also critical for performance. The joint alignment mechanism aligns the exoskeleton with the elbow in a two-dimensional plane but not in a three-dimensional space. Although the exoskeleton is well-aligned with the elbow joint at the beginning of the task, the exoskeleton might slip sideways and be misaligned if the straps are not sufficiently tight. This misalignment in a three-dimensional space can cause the negative or non-significant effect observed from the result of *Bi* for *P*1 and *P*2. To avoid this misalignment, tightening the strap with proper force is important. However, if the strap is excessively tight, it can also cause abnormal muscle activation observed from the result for *P*1. For *P*1, *Tr* was more activated when wearing the exoskeleton in the lifting and holding motions, for which *Tr* is not responsible. This could be because the exoskeleton's cuff and straps compressed *Tr* unevenly and intensively. In future studies, we will modify the cuff design to distribute the pressure on the arm and conduct a parametric study to optimize the strap tightening force that holds the exoskeleton in position and does not disturb muscles.

**Figure 9:**
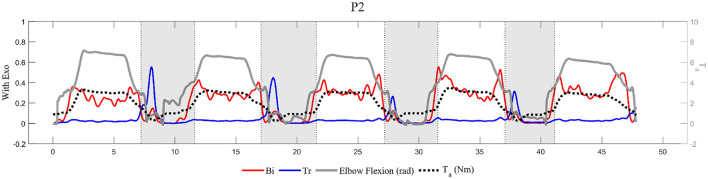
Representative data for participant, *P*2, with a 7 *kg* load. The gray area is where *P*2 extended the elbow by overcoming the resistance caused by the exoskeleton.

Finally, there is ensuring the natural movement of the elbow extension. After the experiment, all participants noted that the exoskeleton seemed to lock or, at least, disturb the elbow extension when lowering a load. This phenomenon is shown in Figure 9, quantitatively. Participant, *P*2, extended their elbow by overcoming the resistance caused by the exoskeleton (gray area) after placing the box on the table. The activation level of *Tr* peaked when extending the elbow, which means that the torque generated by the exoskeleton acted as resistance and required more muscle effort to extend the elbow. Furthermore, the reduction in the elbow flexion angle (Figure 8) shows that the elbow was unnaturally less extended when wearing the exoskeleton. To compensate for the reduced elbow extension, the participants moved other joints, such as the waist or the knee. These “unnatural movements” are not, however, necessarily unhealthy behaviors. The ROM, when wearing the exoskeleton, is still in the safe ROM of the elbow, and the resistance prevents over-extension of the elbow. Moreover, the elbow joint is relatively weaker than the waist or the knee, hence the “unnatural movement” can lead users to distribute the load on the elbow to other joints. As a result, the resistance can help keep the elbow in the safe ROM and distribute the load on the elbow, especially when carrying heavy loads. Despite these advantages, it is necessary to optimize the level of resistance since the muscle activation level is increased and users might feel uncomfortable due to the increase in whole-body energy consumption. In future studies, a parametric study will be conducted to identify the optimal level of resistance that protects the elbow and minimizes whole-body energy consumption. The result will be used to design a user interface that customizes the level of resistance and comfort.

In conclusion, the Elbow-sideWINDER with its novel elbow joint alignment mechanism and medium-level controller seems to provide significant beneficial effects in reducing the activation level of muscles that are responsible for elbow flexion. In particular, when slowly lowering a load using a well-calibrated assistive mode, there was a reduction in the activation level of *Bi* of up to 38.8%. Even when the assistive torque was not perfectly suited to the target load, as occurred with the 7 *kg* load, the activation level of *Bi* decreased by up to 25.7%. In addition, although the exoskeleton disturbed the elbow extension by less than ~13.0°, the activation level of *Tr* also decreased by up to 37.0% when wearing the exoskeleton. These encouraging results show that this exoskeleton is also helpful in stabilizing the elbow joint during dynamic movements. Beyond these positive effects of the Elbow-sideWINDER, this study identifies further studies to enhance its performance and usability, such as optimizing the control gain and level of resistance to maximize assistance and minimize whole-body energy consumption. Finally, this result presents not only the effectiveness of the Elbow-sideWINDER but also important clues to developing elbow exoskeletons for industrial application.

## Data availability statement

The raw data supporting the conclusions of this article will be made available by the authors, without undue reservation.

## Ethics statement

The studies involving human participants were reviewed and approved by Ethical Committee of Liguria (protocol reference number: CER Liguria 001/2019) and complies with the Helsinki Declaration. Written informed consent for participation was not required for this study in accordance with the national legislation and the institutional requirements. Written informed consent was obtained from the individual(s) for the publication of any potentially identifiable images or data included in this article.

## Author contributions

DP built the hardware and control strategy, designed and conducted the experiment, analyzed the result, and drafted the manuscript. CD reviewed the ethical approval. MS modified the experimental protocol. DC and JO supervised the study and provided technical comments on the experiment. All authors collectively revised the manuscript, contributed to the article, and approved the submitted version.
